# Relative age effect and development pathways in French basketball: from youth training structures to senior national teams

**DOI:** 10.3389/fspor.2026.1893863

**Published:** 2026-08-10

**Authors:** Yannis Irid, Audrey Difernand, Jean-François Toussaint, Adrien Sedeaud

**Affiliations:** 1 IRMES-UMR 7329, Institut de Recherche Médicale et d’Épidémiologie du Sport, Université Paris Cité, Institut National du Sport, de l’Expertise et de la Performance (INSEP), Paris, France; 2 Fédération Française de Basketball, Paris, France; 3 Centre d’Investigation en Médecine du Sport, Assistance Publique—Hôpitaux de Paris, Paris, France

**Keywords:** athlete pathways, basketball, relative age effect, talent development, talent identification, youth sport

## Abstract

The relative age effect refers to the advantage experienced by athletes born earlier in the selection year and has been widely documented in youth basketball. However, its influence on long-term development pathways leading to senior elite performance remains insufficiently understood. The aim of this study was to examine the distribution of birth quarters across elite basketball development structures in France and to analyze how relative age relates to progression toward senior national team selection in male and female players. Birth quarter distributions were analyzed for players involved in regional training centers (females = 2,399; males = 2,572), national training centers (females = 157; males = 216), professional club academies (females = 1,981; males = 3,103), and French national teams from youth to senior level (female = 896; male = 803). These distributions were compared with national birth statistics using chi-square (*χ*^2^) tests. In addition, developmental pathways to senior national teams were reconstructed using prospective and retrospective approaches in order to describe patterns of progression across the talent development system. The results showed a strong overrepresentation of players born early in the selection year at the initial stages of elite development for both males and females (*p* < 0.001). The magnitude of this imbalance appeared attenuated at senior international level, where no significant relative age effect was observed (*p* > 0.05). Despite substantial attrition across development stages, distinct progression patterns were observed between male and female players, highlighting differences in how athletes access and move through elite pathways. These findings indicate that relative age strongly influences early selection in elite basketball but does not determine long-term access to senior international performance.

## Introduction

In the competitive landscape of basketball, the identification and long-term development of young athletes are central challenges for clubs, federations, and national team programs. Basketball performance emerges from a complex interaction between physical, technical, cognitive, and psychological factors, all of which evolve dynamically throughout adolescence and early adulthood (1, 2). Consequently, early stages of talent identification and development play a decisive role in shaping future performance trajectories, particularly in structured youth systems where selection decisions often determine access to high-level training environments (3, 4). However, these selection processes are not neutral.

One of the most widely documented sources of bias in youth sport systems is the Relative Age Effect (RAE), which refers to the overrepresentation of athletes born earlier in the selection year within age-grouped competitions (5). In basketball, as in many team sports, athletes born closer to the cut-off date may benefit from temporary advantages in physical maturation, anthropometry, and motor coordination, which can influence coaches' perceptions of talent and readiness for higher levels of competition (6, 7). As a result, relatively younger athletes often face reduced playing opportunities, delayed access to elite training structures, and higher risks of dropout, despite possessing comparable or even superior long-term potential (8, 9).

The presence of RAE has been consistently reported across basketball contexts, including youth academies, national teams, and international competitions (10–15). Previous studies have suggested that the RAE may influence not only selection likelihood but also short-term competitive outcomes, including playing time, efficiency ratings, and team success in youth categories (8, 9). In France, Delorme and Raspaud (6) demonstrated a marked RAE across male and female youth basketball categories, particularly during pubertal years, suggesting that structural selection biases persist throughout key developmental stages. However, findings remain inconsistent across studies, with some reporting clear performance-related advantages for relatively older athletes, whereas others have observed limited or no meaningful differences depending on the competitive context, age category, or performance indicators considered (16–18).

Interestingly, several studies have also reported that the magnitude of RAE tends to decrease, or even disappear, at senior elite levels (13, 19). This apparent attenuation has given rise to alternative interpretations, such as the “*reversal RAE effect*” or the “*underdog hypothesis*,” which propose that relatively younger athletes who survive early selection pressures may develop superior psychological resilience, adaptability, and long-term performance capacities (20, 21), which enables them to improve the transition rate from youth to senior categories, ultimately leading to a more balanced birth-date distribution within senior teams. From this perspective, RAE should not be considered solely as a selection bias, but rather as a dynamic factor that interacts with athletes' developmental pathways over time.

Despite growing interest in these mechanisms, much of the existing RAE literature in basketball remains cross-sectional and descriptive, focusing primarily on birth date distributions at isolated competitive levels. Far fewer studies have examined how RAE shapes longitudinal development pathways, from entry into elite training structures to eventual access to senior international performance. Moreover, comparisons between male and female pathways remain limited, even though structural, competitive, and sociocultural differences may influence selection dynamics and progression patterns differently across genders.

Therefore, the purpose of this study was twofold. First, we aimed to examine the distribution of birth quarters among male and female basketball players across the main elite development structures in France, including regional training centers, national training centers, professional club academies, and national teams, and to compare these distributions with those of the general population. Second, beyond documenting the presence of RAE, we sought to reconstruct and analyze the developmental pathways leading from youth training structures to senior national teams, using both bottom-up (conversion rates) and top-down (insertion rates) approaches. By integrating relative age distributions with pathway analyses, this study aims to provide a more comprehensive understanding of how early selection biases interact with long-term performance trajectories in French basketball.

## Materials and methods

### Ethics

This study was conducted in accordance with the ethical standards of the Declaration of Helsinki. All data were provided by the French Basketball Federation in an anonymized format and handled in compliance with the General Data Protection Regulation of the European Union. The study protocol was reviewed and approved by the scientific committee of the Institute of Medical Research and Sport Epidemiology and by the Scientific, Medical, and Training Council of the French Institute of Sport (INSEP).

### Data sources and study population

Four independent databases were used for the present study: (i) regional training centers, (ii) national training center, (iii) professional club training centers, and (iv) French national teams. Together, these databases allowed a comprehensive reconstruction of the elite basketball development pathway in France.

Since the 1983–1984 season, regional training centers have included athletes from the U13, U14, and U15 age categories. The national training center and professional club training centers (since the 2010–2011 season) encompass athletes from the U16, U17, and U18 categories. While both structures target similar age groups, the national training center operates under the authority of the federation, whereas professional club training centers are managed by private clubs.

The national team database included all players selected for French national teams at the U16, U17, U18, U19, U20, and senior levels. Players were considered uniquely within each national team category. Consequently, the same athlete could appear across multiple age categories (e.g., U16, U18, senior) if selected at different stages of their career progression. Pathway analyses were conducted using the unique athlete identification number in order to reconstruct longitudinal trajectories across development structures. For each athlete, the following variables were available: unique athlete identification number, date of birth, sex, competitive season, and institutional affiliation.

### Birth quarter classification and reference population

Players' dates of birth were categorized into four birth quarters (BQ) according to the calendar year: Q1: January–March; Q2: April–June; Q3: July–September; Q4: October–December. To assess the presence of a relative age effect, birth quarter distributions of basketball players were compared with those of the general French population. Population-level birth data were obtained from the French National Institute of Statistics and Economic Studies (22), based on national birth records over the previous ten years. The mean number of births per quarter across this period was used as the reference distribution. This approach was chosen to provide a stable and representative population baseline, given the extended temporal span of the athlete cohorts included in the study.

### Statistical analysis of relative age effect

Birth quarter distributions were examined separately for males and females across all training structures and national team categories. Chi-square (*χ*^2^) goodness-of-fit tests were used to determine whether observed birth quarter distributions differed significantly from those expected based on the general population reference. Additional descriptive comparisons between youth development structures and senior national teams were illustrated graphically to contextualize the evolution of birth quarter distributions across development levels.

Statistical significance was set at *p* < 0.05. When *χ*^2^ tests were not significant, birth quarter distributions were considered comparable to those of the general population, indicating the absence of a detectable relative age effect at that level. Cramer's V values were interpreted as trivial (<0.10), small (0.10–0.30), medium (0.30–0.50), and large (>0.50), according to Cohen's recommendations (23).

### Pathway analysis

To investigate developmental trajectories beyond cross-sectional relative age distributions, two complementary pathway analyses were conducted separately for male and female players.

First, a prospective (bottom-up) approach was used to examine the proportion of athletes transitioning from regional training centers to higher levels of the development system, including professional club training centers, the national training center, and national teams. This analysis allowed estimation of conversion rates across successive stages of the pathway.

Second, a retrospective (top-down) approach was applied, starting from the senior national teams and tracing athletes' prior involvement in youth national teams and training structures. This method provided insight into insertion rates and the relative contribution of different development pathways to senior international performance. For pathway reconstruction, athletes were considered to have transitioned to a given development structure if they were identified at least once within that structure during the observation period. Consequently, transitions reflected participation at any stage rather than the duration of involvement or the number of seasons spent within each structure. Both approaches were descriptive in nature and aimed to identify structural patterns of progression rather than causal relationships.

### Methodological considerations

Given the retrospective and descriptive design of the study, no causal inferences were drawn regarding the influence of relative age on individual career outcomes. Instead, the analyses were intended to characterize population-level selection patterns and developmental pathways within the French basketball system. All statistical analyses were performed separately for male and female athletes to account for potential gender-specific differences in selection dynamics and development structures.

## Results

### Birth quarter distributions across development structures

The BQ distributions for female and male basketball players across the different development structures are presented in Tables 1, 2, respectively.

**Table 1:** Birth quarter distribution among female French basketball players across development structures and national team categories.

Categories	Q1 (%)	Q2 (%)	Q3 (%)	Q4 (%)	Total	*χ* ^2^	*P*-value	Cramer's V
General population	180 502 (24.83)	177 602 (24.43)	184 609 (25.32)	184 905 (25.43)	727079	—	—	
Regional	919 (38.31)	665 (27.72)	459 (19.13)	356 (14.84)	2399	328.35	<0.001	0.21 (small)
National	67 (42.68)	42 (26.75)	22 (14.01)	26 (16.56)	157	33.28	<0.001	0.27 (small)
Clubs	686 (34.63)	538 (27.16)	405 (20.44)	352 (17.77)	1981	147.05	<0.001	0.16 (small)
U16	96 (36.92)	80 (30.77)	40 (15.38)	44 (16.92)	260	37.14	<0.001	0.22 (small)
U17	20 (28.99)	22 (31.88)	17 (24.64)	10 (14.49)	69	5.31	0.15	0.16 (small)
U18	98 (42.79)	59 (25.76)	30 (13.10)	42 (18.34)	229	47.98	<0.001	0.26 (small)
U19	37 (42.53)	16 (18.39)	15 (17.24)	19 (21.84)	87	14.96	<0.001	0.24 (small)
U20	70 (36.08)	46 (23.71)	36 (18.56)	42 (21.65)	194	14.53	<0.001	0.16 (small)
Seniors	17 (29.82)	17 (29.82)	11 (19.30)	12 (21.05)	57	2.5	0.48	0.12 (small)

**Table 2:** Birth quarter distribution among male French basketball players across development structures and national team categories.

Categories	Q1 (%)	Q2 (%)	Q3 (%)	Q4 (%)	Total	*Χ* ^2^	*P*-value	Cramer's V
General population	180502 (24.83)	177602 (24.43)	184609 (25.32)	184905 (25.43)	727079	—	—	
Regional	1129 (43.90)	706 (27.45)	468 (18.20)	269 (10.46)	2572	664.615	<0.001	0.29 (small)
National	97 (44.91)	63 (29.17)	31 (14.35)	25 (11.57)	216	63.64	<0.001	0.31 (medium)
Clubs	1128 (36.35)	877 (28.26)	626 (20.17)	472 (15.21)	3103	344.606	<0.001	0.19 (small)
U16	117 (45.53)	66 (25.68)	51 (19.84)	23 (8.95)	257	75.013	<0.001	0.31 (medium)
U17	23 (34.85)	25 (37.88)	17 (25.76)	1 (1.52)	66	22.409	<0.001	0.34 (medium)
U18	55 (35.95)	38 (24.84)	41 (26.80)	19 (12.42)	153	17.95	<0.001	0.20 (small)
U19	19 (48.72)	11 (28.21)	6 (15.38)	3 (7.69)	39	15.54	<0.001	0.36 (medium)
U20	70 (36.27)	49 (25.39)	49 (25.39)	25 (12.95)	193	22.07	<0.001	0.20 (small)
Seniors	20 (21.05)	26 (27.37)	28 (29.47)	21 (22.11)	95	1.94	0.58	0.08 (trivial)

Among female players, a marked overrepresentation of athletes born in the first half of the year (Q1–Q2) was observed across the main youth training structures. Specifically, 66.03% of players in regional training centers, 69.43% in the national training center, and 61.79% in professional club training centers were born in Q1 or Q2. In contrast, the general population displayed a relatively uniform distribution across birth quarters. For all three youth training structures, the observed distributions differed significantly from those of the general population (*p* < 0.001).

At the national team level, birth quarter distributions among female players showed variability across age categories. While significant deviations were observed in U16, U18, U19, and U20 female national teams, no significant differences were observed for U17 or senior national teams (*p* > 0.05). Notably, the birth quarter distribution of senior female players did not differ significantly from that of the general population (*p* = 0.48).

Among male players, a similar but more pronounced pattern was observed at youth levels. In regional training centers, 71.35% of players were born in the first half of the year, with a strong overrepresentation of Q1 births (43.90%). Comparable patterns were found in the national training center (74.08% born in Q1–Q2) and professional club training centers (64.61% born in Q1–Q2). For all three structures, birth quarter distributions differed significantly from those of the general population (*p* < 0.001).

At the national team level, male players also displayed significant deviations from the population reference from U16 to U20 (all *p* < 0.001), whereas no significant difference was observed at senior level (*p* > 0.05). The birth quarter distribution of senior male players did not differ from that of the general population (*p* = 0.58).

### Evolution of birth quarter distribution across development levels

**Figure 1:**
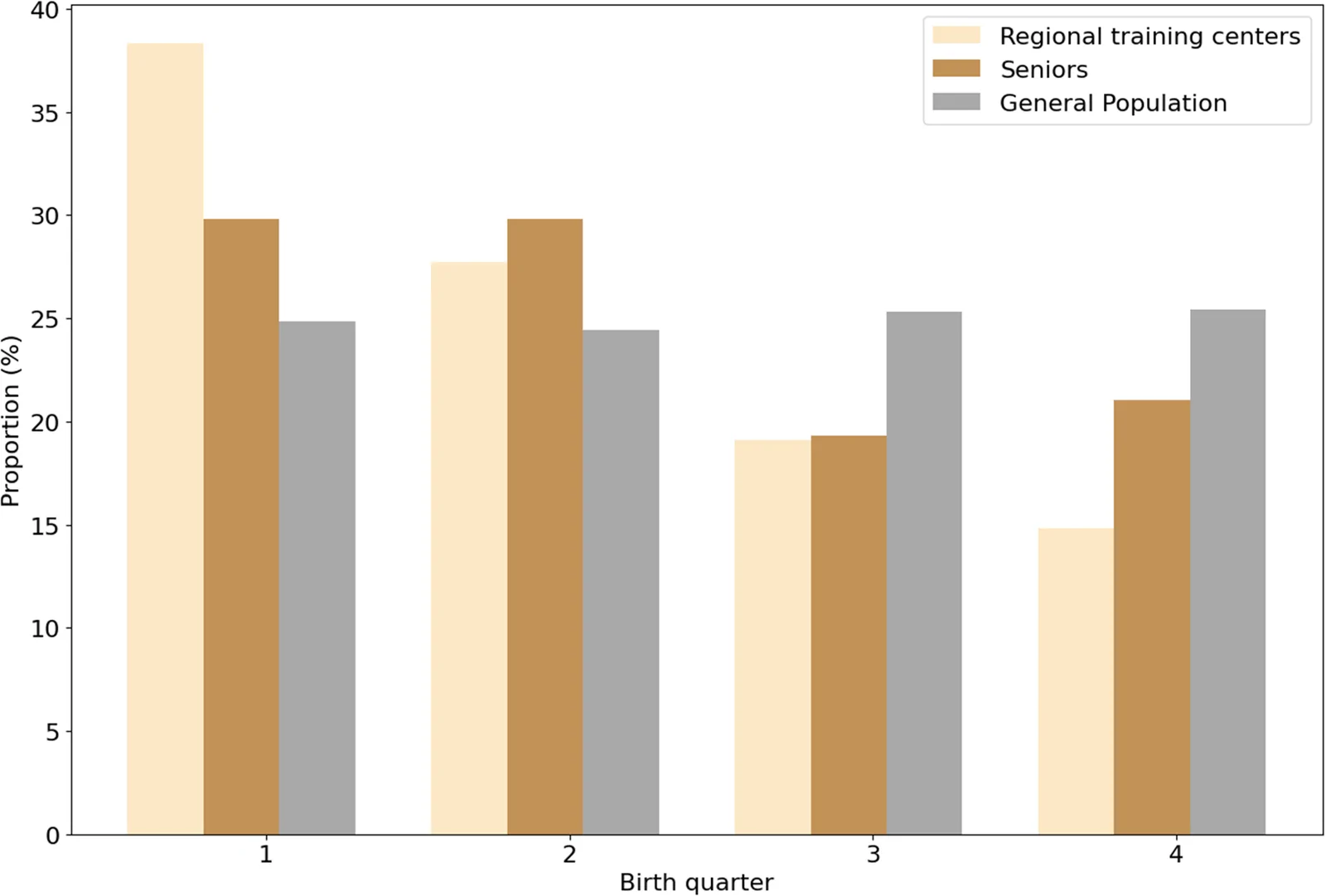
Comparison of birth quarter distributions between female players in regional training centers and senior national teams.

**Figure 2:**
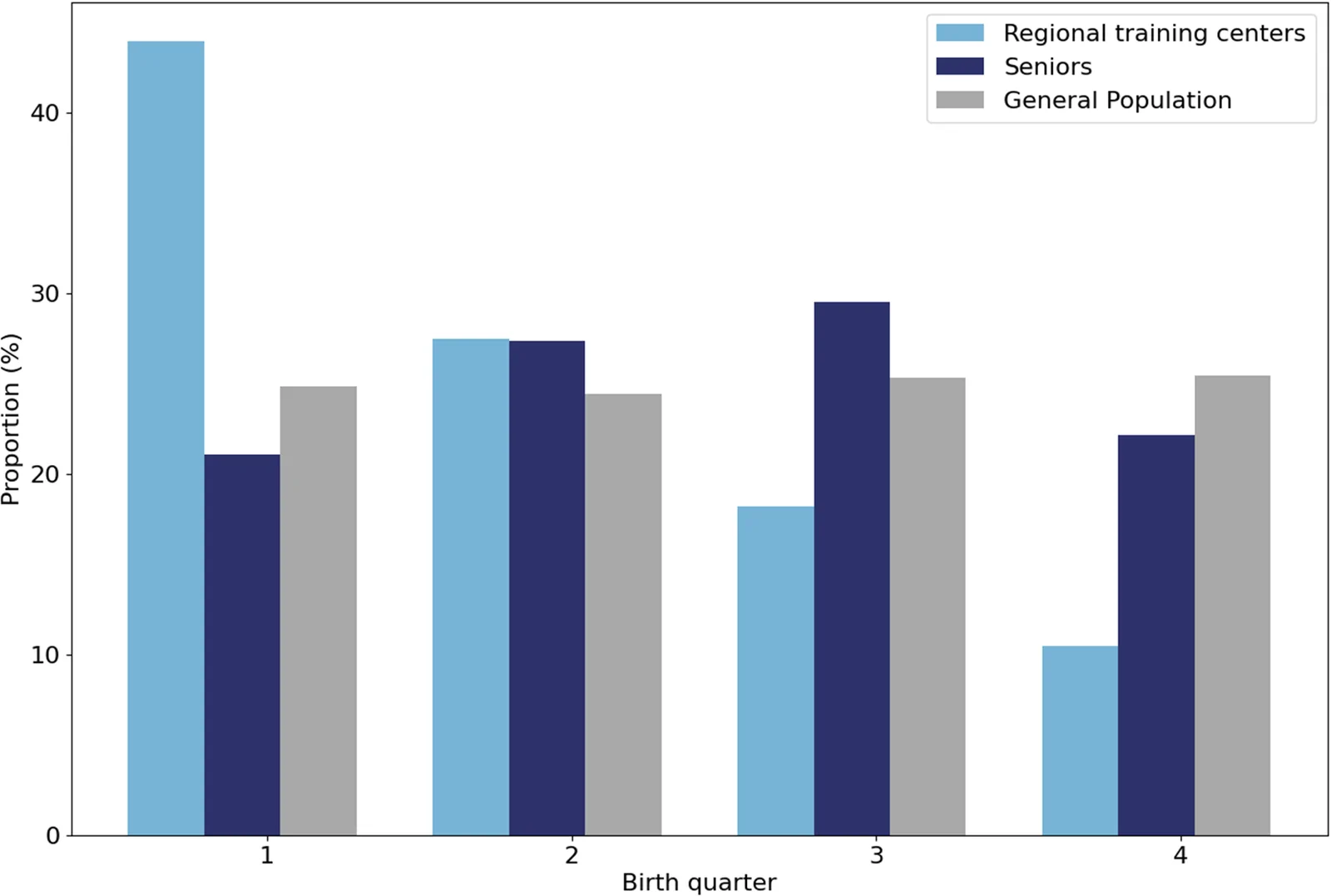
Comparison of birth quarter distributions between male players in regional training centers and senior national teams.

Figures 1, 2 illustrate the comparison of birth quarter distributions between youth development structures and senior national teams for female and male players, respectively.

For female players, the birth quarter distribution in regional training centers differed significantly from that of the general population (*χ*^2^ = 328.35, *p* < 0.001). However, no significant differences were observed between the senior national team and the general population (*χ*^2^ = 2.5, *p* = 0.48).

For male players, regional training centers exhibited a significantly different birth quarter distribution compared with the general population (*χ*^2^ = 664.615, *p* < 0.001). In contrast, the birth quarter distribution of senior male players did not differ significantly from that of the general population (*χ*^2^ = 1.94, *p* = 0.58).

### Development pathways to senior national teams

**Figure 3:**
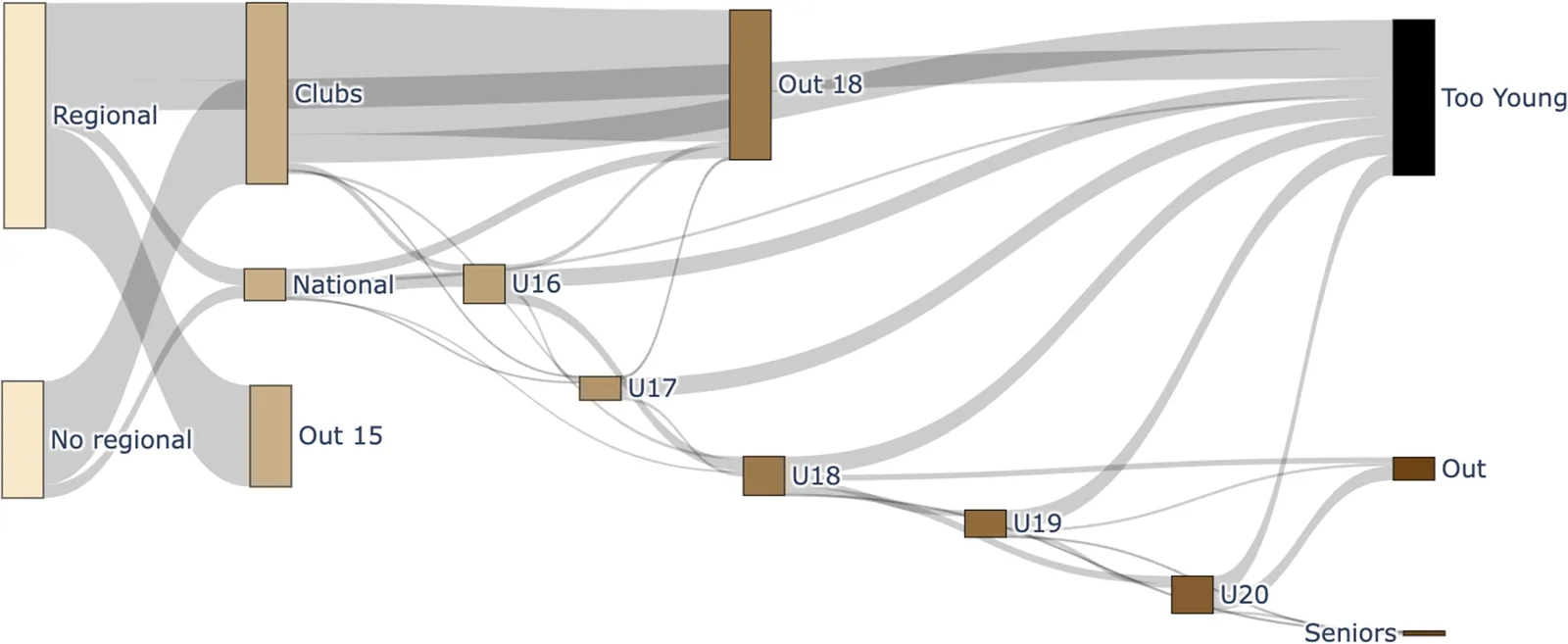
Development pathways from regional training centers to the senior national team in female basketball players.

**Figure 4:**
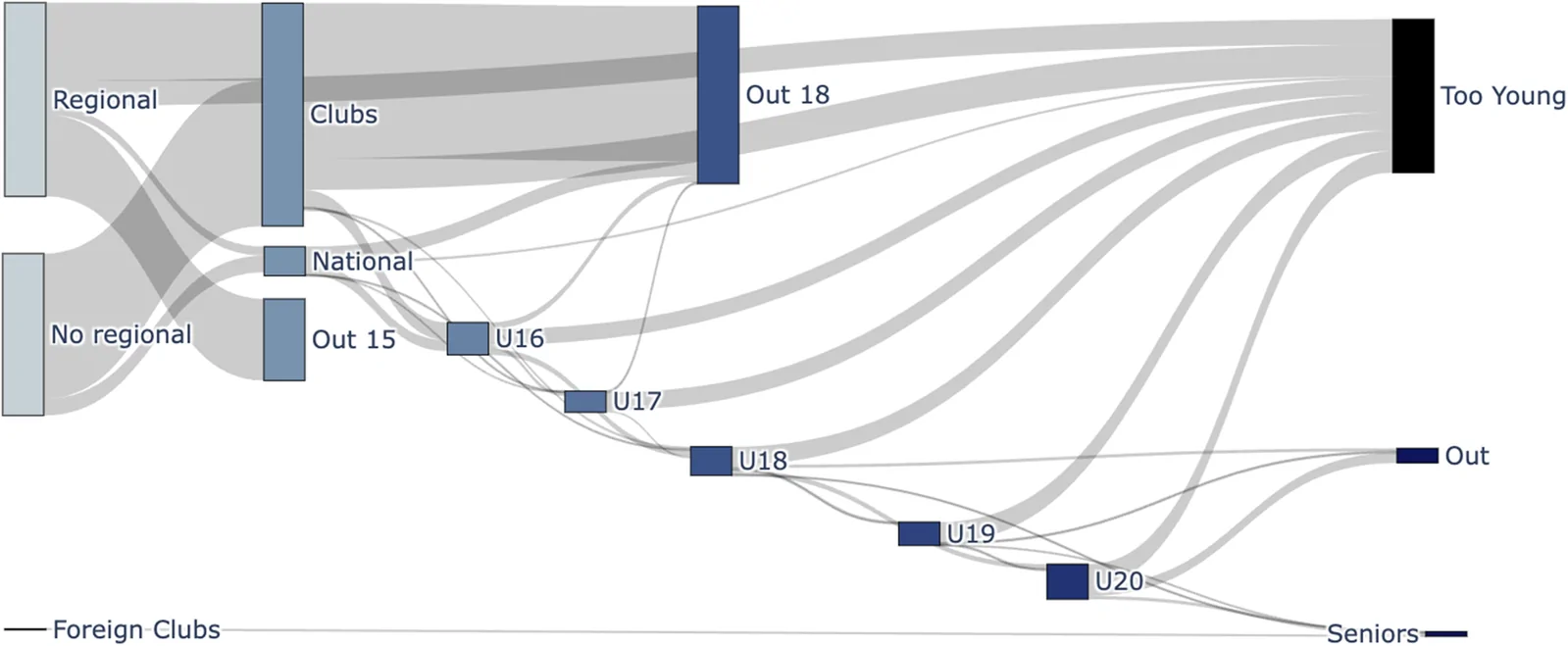
Development pathways from regional training centers to the senior national team in male basketball players.

The developmental pathways leading to senior national team selection are presented separately for male and female players in Figures 3, 4.

#### Prospective (bottom-up) pathway analysis

Among male players originating from regional training centers, 42.07% transitioned to professional club training centers, 4.86% progressed to the national training center, and 57.93% exited the elite development pathway. Following the national training center stage, 48.88% of players were selected at least once for a national team, whereas only 13.08% of those progressing through professional club training centers reached national team selection. Among players who did not pass through regional training centers, 3.0% were eventually selected for the senior national team.

Among female players, 35.37% of athletes from regional training centers transitioned to professional club training centers, 7.54% progressed to the national training center, and 59.69% exited the elite pathway. From the national training center, 63.78% of players were selected for a national team, compared with 8.43% of those emerging from professional club training centers. Among players who did not pass through regional training centers, 1.6% were selected for the senior national team.

#### Retrospective (top-down) pathway analysis

Among female senior national team players, 21.05% had not progressed through regional or national training centers, whereas 64.91% originated from regional training centers. In terms of youth international experience, 14.04% had been selected once for a youth national team, 26.32% twice, and 50.88% at least three times. Professional club training centers were composed of approximately 40% of players originating from regional training centers, with the remainder having developed exclusively within club environments.

Among male senior national team players, 49.47% had not progressed through regional or national training centers, while 35.79% originated from regional training centers. Regarding youth national team experience, 24.21% had been selected once, 30.53% twice, and 16.84% at least three times. Professional club training centers consisted of approximately one-third of players from regional training centers, with the remainder having developed outside of formal academy pathways.

## Discussion

### Relative age effect in French basketball

The first aim of this study was to examine the distribution of birth quarters among male and female basketball players across the main elite development structures in France. The results provide clear evidence of a pronounced RAE at the early stages of talent identification, particularly within regional training centers, national training centers, and professional club academies. In all genders, athletes born in the first half of the selection year were substantially overrepresented compared with the general population, confirming that relative age remains a powerful selection factor in French basketball. These findings are consistent with previous research documenting the presence of RAE in basketball across different competitive levels (8–10, 24, 25). Compared with earlier French data reported by Delorme and Raspaud (6), the magnitude of the effect appears to remain substantial, and possibly greater in some development structures, than that previously reported in French basketball. In their study, 54.17% of female and 52.82% of male U15 players were born in the first half of the year (6). In the present study, these proportions reached 66.03% for females and 71.35% for males in regional training centers. Similarly, in U18-equivalent structures, Delorme and Raspaud reported approximately 52%–54% born in the first half of the year, whereas our study found values exceeding 60% and reaching more than 70% in some male development structures. This comparison suggests that the RAE has persisted and may even have intensified over time within the French basketball development system. Comparable patterns were observed in rugby union. Kelly et al. reported that 42.5% of U15 regional academy players were born in the first quarter compared with only 9.6% in the last quarter (26). In the present study, 43.9% of male and 38.31% of female players in regional training centers were born in the first quarter, whereas only 10.46% and 14.84%, respectively, were born in the fourth quarter. Such parallels across sports reinforce the interpretation that relative age operates as a structural selection bias. Although relatively older athletes may, in some cases, also benefit from more advanced biological maturation, RAE and maturation selection biases represent related but distinct phenomena that should not be considered interchangeable (27, 28). While maturation-associated advantages in anthropometry, strength, and neuromuscular development may contribute to selection decisions during adolescence (7, 29), several additional mechanisms, including accumulated experience, cognitive development, and social influences, have also been proposed to explain relative age effects. However, although the RAE remained evident across most youth national team categories, no significant difference in birth quarter distribution was observed at senior national team level for either males or females. Similar attenuation has been reported in international basketball (25, 30), suggesting that early relative age advantages lose their relevance as biological maturation differences diminish. The reduction of the effect across age categories aligns with the “*reversal RAE effect*” described by McCarthy and colleagues and the “*underdog hypothesis*” proposed by Gibbs et al.. These frameworks suggest that relatively younger athletes who persist through early selection disadvantages may develop enhanced psychological resilience, adaptability, and motivation (20, 21, 31). Nevertheless, it should be noted that the female U17 and senior teams, included relatively small sample sizes. Consequently, the absence of statistically significant differences should be interpreted with caution, as limited statistical power may have reduced the ability to detect subtle relative age effects.

Another complementary explanation for the attenuation of the RAE at senior level relates to survival bias and cumulative cohort attrition. Athletes who successfully progress through highly selective development systems despite early relative age disadvantages may represent a particularly resilient subgroup characterized by exceptional technical, psychological, or motivational qualities (31). Conversely, many relatively older athletes who initially benefited from selection advantages may subsequently leave the pathway due to injury, reduced progression, or increasing competitive demands (32). Consequently, the absence of a detectable RAE among senior players likely reflects the cumulative effects of selective retention and progressive attrition throughout the development pathway rather than the disappearance of the initial selection bias. In this context, the absence of RAE at senior level should not be interpreted as evidence of unbiased selection processes, but rather the cumulative outcome of selection, attrition, and long-term adaptation processes. Early selection may privilege temporarily advanced athletes, yet long-term success appears increasingly determined by developmental progression rather than initial relative age advantages.

### Development pathways and the paradox of early success

Beyond documenting birth quarter distributions, this study reconstructed development pathways from regional training centers to senior national teams using both bottom-up and top-down approaches. Substantial drop-out was observed at each stage, with more than half of athletes exiting the elite pathway before reaching higher structures. Notably, the vast majority of players in professional training centers did not ultimately reach national youth or senior teams. Despite this attrition, the progression patterns observed in French basketball differ from those reported in several other sports. Among senior female players, 71.93% originated from the U18 category, compared with 37.89% among males. These values are considerably higher than those reported by Güllich et al., who observed that only 10%–30% of high-level senior athletes had previously competed at international youth levels (33). Similarly, in football, Brustio et al. reported transition rates between 10% and 20% from youth to senior national teams (34). Conversely, some findings align more closely with Li et al., who showed that a substantial proportion of elite tennis players achieved success at junior level (35). However, when both insertion (top-down) and conversion (bottom-up) rates are considered, as demonstrated by Brouwers et al., only a small fraction of youth elite performers ultimately reach senior top-level status (36). These contrasting findings illustrate the “*early success paradox*,” whereby early achievement may both reflect genuine potential and overestimate long-term predictability (37). In French basketball, the relatively high conversion rates from youth international teams to senior level, particularly among females, may reflect a structured and coherent development system. However, they may also reflect strong early filtering, whereby only the most robust athletes remain within the pathway. Importantly, athletes who are not selected may have limited access to the same developmental environments, coaching quality, resources, and exposure to international competition. This reduced developmental support may constrain their progression through the talent pathway and ultimately decrease their probability of reaching the highest level of performance. Importantly, the coexistence of high youth-to-senior continuity with the disappearance of the RAE underscores the need to distinguish between developmental efficiency and developmental fairness.

The parallel examination of male and female pathways revealed structural differences. Female senior internationals were more likely to have progressed through federation-controlled structures, suggesting a more linear pathway. In contrast, nearly half of male senior internationals did not originate from regional or national training centers, indicating greater pathway heterogeneity. These observed differences may reflect participation depth, competitive density, and structural organization within the respective systems (38). In more competitive male environments, alternative pathways may allow late developers to re-enter elite trajectories at later stages. In female basketball, where pathways appear more centralized, early selection decisions may carry greater long-term consequences.

### Limitations and future directions

Several limitations should be acknowledged. First, the study relied on birth quarter distributions and descriptive pathway analyses, which do not allow causal inference regarding individual career outcomes. In addition, the duration of athletes' involvement within each development structure was not available and therefore could not be incorporated into the pathway analyses. Consequently, transitions were defined according to participation rather than exposure time, which may have provided additional insight into developmental trajectories. Second, data on biological maturation, training load, injury history, and psychosocial factors were not available, despite their known influence on athlete development. Third, the findings are specific to the French basketball system and may not generalize to countries with different organizational structures or cultural contexts. Future research should adopt longitudinal designs integrating physiological, psychological, and contextual variables to better understand how relative age, biological maturation, training history, and psychosocial factors interact throughout athlete development. Such approaches would provide valuable insights into how talent systems can balance performance efficiency with developmental equity.

## Conclusion

This study demonstrates a pronounced RAE across the early stages of elite basketball development in France, with an overrepresentation of athletes born in the first half of the selection year in most youth development structures and national team categories for both males and females. Although some exceptions were observed (e.g., the female U17 national team), the overall pattern indicates that the magnitude of the RAE progressively decreases with age and is no longer evident at the senior international level. The absence of a RAE among senior players does not indicate unbiased early selection processes, but rather reflects the cumulative influence of selection and attrition across development pathways. Relative age acts as a contextual filter shaping access to training environments, without determining long-term performance outcomes. These findings highlight the importance of talent development models that prioritize long-term progression and flexibility in selection processes, in order to reduce the unintended exclusion of late-born athletes. From a practical perspective, these findings support talent identification strategies that emphasize long-term athlete development rather than early selection success. Coaches and federations should consider maintaining flexible development pathways, allowing multiple opportunities for entry and progression, and regularly re-evaluating athletes throughout adolescence. Such approaches may help reduce the unintended exclusion of relatively younger athletes while preserving the overall quality and diversity of the future talent pool.

## Data Availability

The data analyzed in this study is subject to the following licenses/restrictions: Restrictions apply to the datasets due to privacy, institutional, and ethical considerations. The data were provided by the French Basketball Federation (FFBB) and contain sensitive athlete-related information that cannot be publicly shared. Access to anonymized data may be considered upon reasonable request to the corresponding author and subject to approval by the FFBB and relevant ethical authorities. Requests to access these datasets should be directed to Requests to access the dataset should be directed to the corresponding author: Yannis Irid French Basketball Federation (FFBB)/IRMES—INSEP yirid@ffbb.com.
